# Associations between objectively measured physical activity, sedentary behaviour and time in bed among 75+ community-dwelling Danish older adults

**DOI:** 10.1186/s12877-020-01856-6

**Published:** 2021-01-14

**Authors:** Li-Tang Tsai, Eleanor Boyle, Jan C. Brønd, Gry Kock, Mathias Skjødt, Lars G. Hvid, Paolo Caserotti

**Affiliations:** 1 Muscle Physiology and Biomechanics Unit, Center for Active and Healthy Ageing, Department of Sports Science and Clinical Biomechanics, University of Southern Denmark, Campusvej 55, Odense M, 5230 Odense, Denmark; 2 Clinical Biomechanics Unit, Department of Sports Science and Clinical Biomechanics, University of Southern Denmark, Odense, Denmark; 3 Centre of Research in Childhood Health, Department of Sports Science and Clinical Biomechanics, University of Southern Denmark, Odense, Denmark; 4 Exercise Biology, Department of Public Health, Aarhus University, Aarhus, Denmark

**Keywords:** Aging, Accelerometer, Physical activity, Mobility

## Abstract

**Background:**

Older adults are recommended to sleep 7–8 h/day. Time in bed (TIB) differs from sleep duration and includes also the time of lying in bed without sleeping. Long TIB (≥9 h) are associated with self-reported sedentary behavior, but the association between objectively measured physical activity, sedentary behavior and TIB is unknown.

**Methods:**

This study was based on cross-sectional analysis of the Healthy Ageing Network of Competence (HANC Study). Physical activity and sedentary behaviour were measured by a tri-axial accelerometer (ActiGraph) placed on the dominant wrist for 7 days. Sedentary behavior was classified as < 2303 counts per minute (cpm) in vector magnitude and physical activity intensities were categorized, as 2303–4999 and ≥ 5000 cpm in vector magnitude. TIB was recorded in self-reported diaries. Participants were categorized as UTIB (usually having TIB 7–9 h/night: ≥80% of measurement days), STIB (sometimes having TIB 7–9 h/night: 20–79% of measurement days), and RTIB (rarely having TIB 7–9 h/night: < 20% of measurement days). Multinominal regression models were used to calculate the relative risk ratios (RRR) of being RTIB and STIB by daily levels of physical activity and SB, with UTIB as the reference group. The models were adjusted for age, sex, average daily nap length and physical function.

**Results:**

Three hundred and fourty-one older adults (median age 81 (IQR 5), 62% women) were included with median TIB of 8 h 21 min (1 h 10 min)/day, physical activity level of 2054 (864) CPM with 64 (15) % of waking hours in sedentary behavior. Those with average CPM within the highest tertile had a lower RRR (0.33 (0.15–0.71), *p* = 0.005) for being RTIB compared to those within the lowest tertile of average CPM. Accumulating physical activity in intensities 2303–4999 and ≥ 5000 cpm/day did not affect the RRR of being RTIB. RRR of being RTIB among highly sedentary participants (≥10 h/day of sedentary behavior) more than tripled compared to those who were less sedentary (3.21 (1.50–6.88), *p* = 0.003).

**Conclusions:**

For older adults, being physically active and less sedentary was associated with being in bed for 7–9 h/night for most nights (≥80%). Future longitudinal studies are warranted to explore the causal relationship sbetween physical activity and sleep duration.

## Background

Sleep is a complex process linked with vital physiological functions such as development, energy conservation, immune response and cognition [1]. Essentially, sleep allows the body to recover from daily activities. Recommended sleep duration differs across lifespan in response to our body’s different stages of physiological development. Adults are recommended to sleep 7–9 h/night while older adults aged ≥65 are suggested to sleep 7–8 h/night [2]. Due to low cost and easy administration, subjective measurements of sleep duration has been widely used in epidemiological studies across different age groups and has been found to be associated with obesity, diabetes, hypertension and mortality [3]. In old age, both insufficient and excessive sleep duration can be detrimental to health [4–6] and physical function [7, 8].

Time in bed (TIB) differs from sleep duration, as it also includes the time of lying in bed without sleeping. Together with sleep duration, TIB can offer important additive information when evaluating the consequences of sleep behaviour on health outcomes [9]. One previous study investigated several different combinations of long and short TIB (≥9 h vs. ≤6 h) and self-reported sleep duration (≥9 vs. ≤6 h) among older adults, and found that those with both long TIB (≥9 h) and long sleep duration (≥9 h) showed greatest decline in physical function followed by those with long TIB (≥9 h) but short sleep duration (≤6 h). Long TIB alone also predicted accelerated decline in objectively measured physical function, including slower walking speed [9]. The underlying mechanism could be a deficiency in the restorative function of sleep or poor sleep quality which means the individual was not well-rested even after prolonged TIB.

Several studies have linked subjective long sleep duration with lower level of physical activity and higher amount of sedentary behavior [9–13]. Among older adults, both short (≤6 h) and long (≥9 h) TIB is associated with sedentary behavior assessed by questionnaires [9]. However, recall bias reduces the reliability for assessing physical activity among older adults with self-reported methods, especially for light-intensity physical activity and sedentary behavior [14]. With the advancement of technology, accelerometers have been widely applied to epidemiological studies and have contributed to increased precision when measuring physical activity and sedentary behavior among community-dwelling older adults [15, 16].

To our knowledge, no study has investigated the association between TIB and objectively measured physical activity among older adults adjusting for functional status. We postulate that a higher prevalence of inadequate TIB (both short and long) could reflect sleep deprivation (short TIB) [4, 5], deficiency in the restorative function of sleep (long TIB) or poor sleep quality (short or long TIB). All the above scenarios can be associated with being less physically active and higher levels of sedentary behaviour in old age [17]. The purpose of this study was to investigate the associations between objectively measured physical activity, sedentary behaviour and time in bed among 75+ years old community-dwelling Danish older adults.

## Methods

### Study design and population

The present study was based on cross-sectional analysis of the Healthy Ageing Network of Competence (HANC) Study’s baseline data [18, 19]. The HANC study received ethical approval from The Regional Scientific Ethical Committees for Southern Denmark (case number S-20120149).

#### The HANC study

The aim of the HANC study was to optimize the preventive home visit by adding objective and subjective assessment tools to evaluate the risk factors of functional impairment and disability in community-dwelling older adults. All community dwelling residents of Denmark aged 75 years and older are offered a preventive home visit by a health care worker from the municipality. Between March 2013 and September 2014, the preventive home visit’s offer letter of Odense Municipality also included information about the HANC study. Residents who took part in the preventive home visit and consented to participate in the HANC study underwent a series of tests assessing physical function, cognitive function, quality of life etc., and wore an accelerometer (tri-axial accelerometer (ActiGraph GT3X/GT3X+; ActiGraph inc, Pensacola, FL, USA)) for 7 days on their dominant wrist. The accelerometer was worn both during waking hours and sleep time (24 h) and was only removed if the participants was showering or engaging in any water activities. Detailed instructions were given on what to record in their accelerometer wear-time diary (e.g., sleep, wake and nap times). They were encouraged to maintain their usual daily routines during the 7-day period.

#### Study population

Among participants of the HANC study, those who agreed to wear the accelerometer for 7 days, who had valid accelerometer data along with completed accelerometer wear-time diary were included in the study.

### Measures

#### Objectively measured physical activity and sedentary behaviour

Physical activity and sedentary behaviour were calculated using the data from the accelerometer. The accelerometer data were first downloaded by the ActiLife software (version 6.4.11) into ActiGraph counts metric (.gt3x file format) and converted into .agd file format without Low Frequency Extension Filter [20]. Thereafter, the data files were analyzed using a 60-s epoch and produced counts per minute (cpm) in vector magnitude by a custom-made software (Propero) developed at the Department of Sports Science and Clinical Biomechanics, University of Southern Denmark. Valid data was defined as those with minimum 10-h recordings for at least 4 days. Periods of 30 min or longer of continuous zero counts allowing for one single spike below 100 counts was defined as non-wear and set as missing in the analysis. TIB of each night was estimated from the self-reported accelerometer wear-time diary as the time from going to bed until getting out of bed the next day. TIB periods was set as zero in the individual accelerometry recordings and thus identified as non-wear. Finally, physical activity and sedentary behavior were summed for the accelerometer wear time between 05:00–24:00 (maximum 19 h/day) and time spent in the defined intensities of physical activity and sedentary behavior were calculated using accelerometer wear time as denominator and expressed in percentage.

Sedentary behavior was defined as 0–2302 cpm in vector magnitude, according to a validated cut-point for wrist-worn accelerometers in older adults with thigh-worn accelerometer (ActivPAL) as reference [21]. Being highly sedentary (≥10 h/day) has been linked to several adverse health indicators, including diminished physical function [22] and increased risk for cardiovascular disease [23]. In this study, highly sedentary was defined as having sedentary behavior of ≥65% of *accelerometer wear time (median 15 h 25 min/day)*, corresponding to 10 h/day in the current study population. There are currently no available cut-points reflecting moderate and vigorous intensity physical activity for wrist-worn accelerometers, and we have pragmatically decided to report activities at relatively higher intensities by ranges of 2303–4999 and ≥ 5000 counts per minute. The purpose of using these categories is to describe physical activity at relatively higher intensities when compared to sedentary behavior. Therefore, they may not be directly comparable to moderate and vigorous intensity physical activity.

#### Time in bed (TIB)

Older adults are recommended to sleep 7–8 h/night [2]. Considering sleep onset latency (the time a person lays in bed before falling asleep) increases with age [24], we deemed TIB of 7–9 h as appropriate for this age group. Participants were categorized according to how frequent they reached 7–9 h of TIB in the measurement days: *UTIB* (usually having TIB 7–9 h/night: ≥80% of measurement days), *STIB* (sometimes having TIB 7–9 h/night: 20–79% of measurement days), and *RTIB* (rarely having TIB 7–9 h/night: < 20% of measurement days).

#### Covariates

Age and sex were obtained by means of a structured interview at the participant’s home. Daily nap length was calculated from the accelerometer wear-time diary. The Short Physical Performance Battery (SPPB) [25] was used to assess lower-extremity physical function during the first home visit. The battery consists of three tests that assess standing balance, walking speed over 3 m, and sit-to-stand from a chair. Each task was rated from 0 to 4 points and added up to produce a SPPB total score (ranges from 0 to 12, with higher score indicating better function).

### Statistical analysis

Participants’ characteristics were described using median and interquartile ranges (IQR) or percentages. Differences between the UTIB, STIB, and RTIB were compared using ANOVA for continuous variables and chi-square tests for categorical variables. Kruskal-Wallis test was used for the same purpose for non-normally distributed variables. Bonferroni adjustment were applied to all Post Hoc Tests.

Two multinominal regression models were used to calculate the relative risk ratios (RRR) of being RTIB and STIB with UTIB as the reference group. Model I was adjusted for age, sex, average daily nap length and SPPB total score and was used to compare the highest and the middle tertile of three measures of physical activity to the lowest tertile: (1) average CPM; (2) % of time in intensity 2303–4999 cpm/day; (3) and % of time in intensity ≥5000 cpm/day. Besides all the covariates of Model I, Model II was additionally adjusted for % in intensity ≥5000 cpm/day to calculate the RRR of being RTIB and STIB for those who were highly sedentary (daily sedentary behavior ≥65% of wear time) compared to those who were not (daily sedentary behavior < 65% of wear time). From the same multinominal regression models, probability of being RTIB, STIB and UTIB by daily levels of physical activity and sedentary behavior were calculated. The models met all assumptions of a multinominal regression model and there were no interactions between age, sex, and the other variables.

Sensitivity analysis (data shown in supplementary file) was conducted to compare the amount of daily physical activity and sedentary behavior after removing sleep duration with two different methods: by a fixed period (23:00–8:00) and by data from accelerometer wear-time diary.

Statistical significance was set at *p* <  0.05. Data were presented as mean ± standard deviation in the descriptive analysis and as relative risk ratios and probability with confidence intervals in the multinominal models. Analyses were conducted in Propero (custom-made in University of Southern Denmark), Microsoft excel, SPSS (version 24; IBM Corp., Armonk, NY, USA), and Stata 16 (StataCorp. 2019. Stata Statistical Software: Release 16. College Station, TX: StataCorp LLC.).

## Results

Five hundred and fifty-four older adults agreed to participate in the HANC study, 482 wore the accelerometer for 7 days, and of these 341 had valid accelerometer data along with completed accelerometer wear-time diary and were included in the study. The included participants of this study (median age 81 (interquartile range (IQR) 5), 62% women) had an median TIB of 8 h 21 min (1 h 10 min) per day, a physical activity level of 2054 (864) cpm/day, and wore the accelerometer 15 h 25 min (1 h 16 min) daily (between 05:00–24:00). Descriptive statistics of UTIB, STIB, and RTIB are shown in Table 1. Compared to UTIB, RTIB were less physically active (1919 (824) vs. 2130 (768) cpm/day, *p* <  0.001), had more sedentary behaviour (67 (15) vs. 62 (14)%, *p* <  0.001), and spent less time in 2303–4999 cpm intensity zone (21 (8) vs. 23 (8) %, *p* <  0.026). Post-hoc analysis on physical activity and sedentary behavior showed that STIB were more active and less sedentary than RTIB, but no difference was observed between STIB and UTIB. UTIB had the highest SPPB score (11 (3)), reflecting better physical function than STIB (10 (3)) and RTIB (9 (4)).

**Table 1:** Descriptive Statistics of UTIB, STIB, and RTIB (*n* = 341)

	UTIB (*n* = 123)	STIB (*n* = 134)	RTIB (*n* = 84)	*p*-value
	Median (IQR)	Median (IQR)	Median (IQR)	
Age (years)	81 (6)	81 (4)	82 (6)	0.445
Women (%)	64	62	60	0.789
TIB (hr + min/night)	8 h 6 min (31 min)	8 h 32 min (1 h 19 min)	9 h 23 min (1 h 26 min)	< 0.001
Nap length (min/day)	43 (60)	36 (60)	41 (65)	0.835
SPPB total score	11 (3)	10 (3)	9 (4)	0.007
Accelerometer wear time (hr + min/day)	15 h 42 min (41 min)	15 h 23 min (1 h 6 min)	14 h 37 min (1 h 9 min)	< 0.001
Average CPM	2130 (768)	2102 (821)	1919 (824)	< 0.001
Highly Sedentary (%)	40	44	66	0.001
% of accelerometer wear time in SB (0–2302)	62 (14)	63 (14)	67 (15)	< 0.001
% of accelerometer wear time in intensity 2303–4999	23 (8)	23 (8)	21 (8)	0.026
% of accelerometer wear time in intensity ≥5000	14 (12)	14 (10)	11 (10)	0.006

Statistical method: Chi square, one-way ANOVA, and Kruskal-Wallis test

*Abbreviations and definitions*: *UTIB* usually having TIB 7–9 h/night: ≥80% of measurement days, *STIB* sometimes having TIB 7–9 h/night: 20–79% of measurement days, *RTIB* rarely having TIB 7–9 h/night: < 20% of measurement days; Highly Sedentary: daily SB ≥ 65% of wear time; *IQR* interquartile range, *TIB* time in bed, *hr* hour, *min* minutes, *SPPB* short physical performance battery, *SB* sedentary behavior, *CPM* counts per minute. Physical activity and sedentary behaviour were calculated in vector magnitude from 05:00–24:00 (19 h)/day excluding TIB

Table 2 shows relative risk ratios (RRR) of being RTIB and STIB by daily levels of physical activity and sedentary behavior with UTIB as reference. Those with average CPM within the highest tertile had lower RRR of being RTIB compared to their counterparts within the lowest tertile (0.33 (0.15–0.71), *p* = 0.005). Higher percentages of physical activity in intensities 2303–4999 and ≥ 5000 did not have statistically significant effect on the RRR of being RTIB. Those who were highly sedentary had an elevated RRR of being RTIB, which more than tripled the corresponding RRR for those who were less sedentary (3.21 (1.50–6.88), *p* = 0.003). Levels of physical activity and sedentary behavior did not affect the RRR of being STIB.

**Table 2:** Relative Risk Ratios for RTIB and STIB by Physical Activity Levels and Sedentary Behavior (*n* = 341)

	RTIB		STIB		Overall *p* value
	RRR (95% CI)	*p* value	RRR (95% CI)	*p* value	Statistical model
**Average CPM**					0.050
Highest tertile	0.33 (0.15–0.71)	0.005	0.84 (0.43–1.63)	0.610	Model I
Middle tertile	0.72 (0.36–1.47)	0.370	1.18 (0.62–2.25)	0.619	
Low tertile	1		1		
**% in intensity 2303–4999**					0.276
Highest tertile	0.53 (0.26–1.08)	0.082	1.16 (0.64–2.14)	0.615	Model I
Middle tertile	0.78 (0.40–1.52)	0.468	1.12 (0.61–2.05)	0.713	
Low tertile	1		1		
**% in intensity ≥ 5000**					0.438
Highest tertile	0.62 (0.28–1.35)	0.227	0.84 (0.43–1.64)	0.605	Model I
Middle tertile	1.18 (0.57–2.44)	0.652	1.30 (0.68–2.49)	0.428	
Low tertile	1		1		
**Sedentary Behavior**					0.005
Highly sedentary	3.21 (1.50–6.88)	0.003	1.07 (0.57–2.00)	0.825	Model II
Not highly sedentary	1		1		

Multinominal logistic regression test with UTIB as reference category. Model I was adjusted for age, sex, average daily nap length and SPPB total score. Model II was additionally adjusted for % in intensity ≥5000

*Abbreviations and definitions*: *UTIB* usually having TIB 7–9 h/night: ≥80% of measurement days, *STIB* sometimes having TIB 7–9 h/night: 20–79% of measurement days, *RTIB* rarely having TIB 7–9 h/night: < 20% of measurement days; Highly Sedentary: daily sedentary behavior ≥65% of wear time, *RRR* Relative Risk Ratios, *95% CI* 95% Confidence Interval, *CPM* counts per minute

**Fig. 1:**
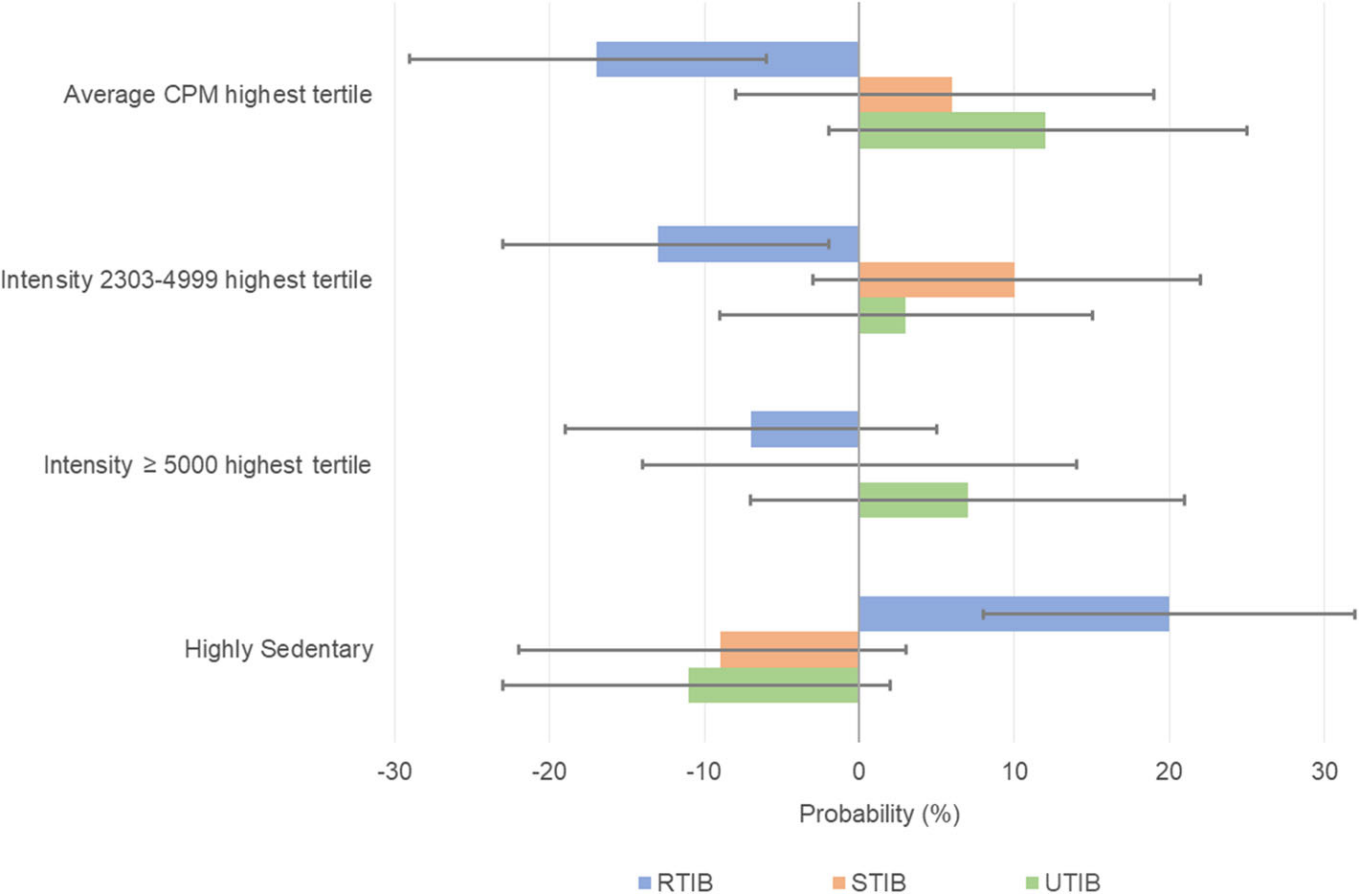
Probability for RTIB, STIB, and UTIB by Physical Activity Levels and Sedentary Behavior (*n* = 341). Abbreviations and definitions: UTIB (usually having TIB 7–9 h/night: ≥80% of measurement days); STIB (sometimes having TIB 7–9 h/night: 20–79% of measurement days); RTIB (rarely having TIB 7–9 h/night: < 20% of measurement days); Highly Sedentary: daily sedentary behavior ≥65% of wear time; RRR = Relative Risk Ratios; 95% CI = 95% Confidence Interval; CPM = counts per minute

Figure 1 depicts the probability for being RTIB, STIB and UTIB by daily levels of physical activity and sedentary behavior. Overall, being more physically active and less sedentary decreases the probability of being RTIB: having average CPM within the highest tertile decreases the probability of being RTIB by 17% (95% CI 6–29%, *p* = 0.003); spending more time in intensity 2303–4999 (within the highest tertile) decreases the probability of being RTIB by 13% (95% CI 2–23%, *p* = 0.023); being highly sedentary increases the probability of being RTIB by 20% (95% CI 8–32%, *p* = 0.001).

Results from the sensitivity analysis (data shown in supplementary file) found a 3% inflation in sedentary behavior when TIB was removed by a fixed period (23:00–8:00) compared to when TIB was removed individually for each day according to the accelerometer wear-time diary.

## Discussion

The results from wrist-worn accelerometers indicate that older adults reporting 7–9 h/night of TIB for ≥80% of nights in a 7-day measurement period were more physically active and less sedentary than those who reported TIB 7–9 h for less than 20% of the measured nights. Those with higher daily CPM were less likely to be RTIB. In contrast, those who were highly sedentary had an elevated relative risk ratio (RRR) of being RTIB, which more than tripled the RRR compared to those who were less sedentary. This coincides with earlier studies which linked highly sedentariness (≥10 h/day) to not achieving 7–8 h/night of sleep for older women [13]. In line with an earlier study, our results suggest that TIB, a relatively easy outcome to collect in epidemiological study, can provide important additive information for evaluating the relationship between sleep behaviour and health outcomes [9].

According to literature, human sleep duration decreases linearly across lifespan, but plateaus after the age of 60 [26]. Our study population was 81 (5) years old and should ideally sleep 7–8 h/night. Taking sleep latency into account, we considered TIB of 7 to 9 h/night as appropriate for this population. We observed that UTIB had shortest median TIB (8 h 6 min (31 min)/night), which increased for STIB (8 h 32 min (1 h 19 min)/night) and RTIB (9 h 23 min (1 h 26 min)/night). More specifically, 76% of RTIB had prolonged TIB (≥9 h/night) while 0% of UTIB had prolonged TIB. This is supported by several earlier studies that linked long sleep duration with lower level of physical activity and higher amount of sedentary behavior [9–13].

There are a few explanations to how being physically active can be linked with good sleep. Regular physical activity can raise body temperature and as the body temperature is regulated later on by thermal homeostasis, feelings of drowsiness can be triggered and help an individual to fall asleep [27]. Also, physical activity can contribute to prolonged time spent outdoors and exposure to natural light, an important element in helping body establish circadian rhythms (sleep-wake cycle) [28].

SPPB is a composite outcome measure of lower limbs physical function with excellent reliability (ICC 0.88–0.92) [29] and is able to predict disability, institutionalization, falls, and mortality among older adults [29–32]. The results of this study showed that UTIB had the highest SPPB score (11 (3)), reflecting better physical function, followed by STIB (10 (3)) and RTIB (9 (4)). This result is consistent with earlier studies linking long TIB to accelerated decline in walking speed and SPPB score [9, 33]. All the multinomial regression models in this study were adjusted for SPPB total score, which confirms that TIB has an independent association with physical activity and sedentary behavior regardless of individual’s physical function.

An interesting finding was that sedentary behavior and average CPM were found to alter relative risk and probability of being RTIB, but not for physical activity at higher intensities (2303–4999 and ≥ 5000 cpm/day), which showed similar trend but did not reach statistical significance. One explanation may be lower sensitivity of wrist-worn accelerometers to distinguish physical activity at higher intensities [34] and lack of existing cut-points for moderate and vigorous activities for older adults. The greater range of motion of the shoulder joint allows for high variability of wrist movement even under sitting position, when the overall physical activity intensity remains relatively low. A recent study which compared the accuracy of accelerometers placed on the hip, thigh, and wrists for measurement of physical activity and sedentary behavior categorized via direct observation found that, accelerometers worn on right wrist had sensitivities and specificities of 93–99% for sedentary behavior and light intensity physical activity but only 67–84% for moderate-to-vigorous physical activity. Sensitivity and specificity values for the thigh- and hip-worn accelerometers were higher than wrist-worn accelerometers, being 87–99% for all physical activity intensity categories [34].

We decided to exclude physical activity and sedentary behavior measured between 24:00–05:00 due to two reasons. First, great majority of participants reported TIB during this period (out of 2048 days of accelerometer wear-time diary entries, we observed 21 separate days (1%) of waking up and 269 separate days (13%) of going to bed between 24:00–05:00). Therefore, even though we may slightly underestimate daily physical activity for these individuals who were awake between 24:00–05:00, it should not have significantly affected our results. Second, the sporadic activities recorded by accelerometers between 24:00–05:00 in this age group could have likely been due to poor sleep quality, reflecting restlessness during TIB or getting in and out of bed and thus should not be pooled into daily physical activity and sedentary behavior for the purpose of this study.

Compared to self-reported sleep duration, TIB could be easier to recall and less suspectable to recall bias, a common problem among older adults. One study comparing self-reported sleep duration and TIB with objective measures of sleep among 35 long sleepers aged 50–70 years discovered that participants tended to over-report their sleep duration. This means they actually had shorter sleep duration than they thought, and long TIB instead of long sleep duration may be more relevant to the increased health risk observed. When feasible, it is recommended for future studies to collect both self-reported sleep duration and TIB as they complement each other and together can provide valuable information about sleep behavior [9].

The strength of this study is the unique sample of very old Danish community-dwelling subjects with objectively assessed physical activity and sedentary behavior over 7 days and that the waking hours of each day for each participant was individually cleaned according to the accelerometer wear-time diary. Sensitivity analysis (data shown in supplementary file) was conducted to compare the amount of daily sedentary behavior after removing sleep duration with two different methods: by a fixed period (23:00–8:00) and by data from accelerometer wear-time diary; and found a 3% inflation in sedentary behavior with the first method due to misclassification of sleep. No previous study has focused specifically on the association between objectively measured physical activity and TIB among older adults. Compared to using subjective measures or pedometers in monitoring physical activity, accelerometers generally show higher sensitivity, with the advantage of differentiating between physical activity intensities. The heterogeneity of our participants in terms of age and gender increases the generalizability of our results among community-dwelling older people.

The study has some limitations that should be taken into consideration when interpreting the results. Accelerometer wear time in our study was self-reported and thus may be subject to reporting bias [35]. However, determining accelerometer wear time by a data-driven method is also prone to bias, especially in highly sedentary older adults as it may be challenging to distinguish whether they are resting, simply being inactive, or if the device has been taken off [35, 36]. Sleep efficiency (the proportion of sleep time within TIB) has been found to be associated with physical activity among older adults [37] and could be a potential cofounder for our study. Without information of sleep efficiency, we cannot determine whether those who laid in bed for the same amount of time gained similar or different amount of sleep, and therefore we cannot distinguish how much health effect is due to TIB and how much is due to sleep. Future studies are warranted to take sleep efficiency into account when investigating the association between TIB and physical activity.

## Conclusion

In conclusion, for older adults over 75 years, being physically active and less sedentary was associated with being in bed for 7 to 9 h/night during most nights (≥80%). Practical implication of this study is to recommend older adults to be in bed for preferably 7 to 9 h per night. In order to achieve this goal, clinicians can recommend evidence-based strategies to older adults for promoting adequate TIB and improving sleep quality. For example, listening to soft music at bedtime [38], reducing caffeine intake and being aware that older adults may be more sensitive to caffeine compared to younger adults [39, 40], evening light exposure [41] and light therapy [42]. Future longitudinal studies are warranted to explore the causal relationship between physical activity, time in bed, and sleep duration. Lower sensitivity of wrist-worn accelerometers to distinguish physical activity at higher intensities needs to be considered and perhaps replaced or coupled with measures from thigh-worn or hip-worn accelerometers.

## Supplementary Information

**Additional file 1.**

## Data Availability

The datasets generated and/or analyzed during the current study are not publicly available due to privacy and confidentiality restrictions pertaining to person-level health information, which contains personal identifiers, in Denmark, governed by General Data Protection Regulation (GDPR). However, the data set creation plan and underlying analytic code are available from the corresponding author on reasonable request.

## Abbreviations

TIB: Time in bed
hr.: Hour
CPM/cpm: ccounts per minute
RRR: Relative Risk Ratio
SPPB: Short physical performance battery
SB: Sedentary behavior
95% CI: 95% Confidence Interval
