# Supplementary material for: Associations between objectively measured physical activity, sedentary behaviour and time in bed among 75+ community-dwelling Danish older adults
Source: BMC Geriatr. 2021 Jan 14;21:53. doi: 10.1186/s12877-020-01856-6 (PMC7807682; doi:10.1186/s12877-020-01856-6)
Supplement: Supplementary file 1 — Additional file 1. [file 12877_2020_1856_MOESM1_ESM.pdf]

Sensitivity analysis was conducted to compare the amount of daily sedentary behavior after removing sleep duration with two different methods: by a fixed period (23:00-8:00) and by data from accelerometer diary; and found a 3% inflation in sedentary behavior with the first method due to misclassification of sleep (highlighted in yellow, Table 3).

Table 3. Time spent in different intensities of physical activity and sedentary behaviour by removing fixed sleep time or individualized sleep time for each day (n=341)

**Protocol of removing sleep time**	**Fixed**	**Individualized**	*P value
	Median (IQR)	Median (IQR)	
Average CPM	1877 (755)	2054 (864)	< 0.001
% of time in SB (0-2302)	67 (14)	64 (15)	< 0.001
% of time in intensity 2303-4999	21 (8)	23 (9)	< 0.001
% of time in intensity ≥ 5000	11 (10)	13 (11)	0.073

Physical activity and sedentary behaviour were calculated in vector magnitude from 05:00-24:00 (19hr)/day; **Fixed sleep time:** remove sleep time as 23:05-07:45; **Individualized sleep time:** by removing sleep time according to sleep log for each day.

*Independent sample t-test and Mann-Whitney U test.
